# Multiculturalism and interculturalism: redefining nationhood and solidarity

**DOI:** 10.1186/s40878-018-0082-6

**Published:** 2018-05-17

**Authors:** Riva Kastoryano

**Affiliations:** Sciences Po – CERI – CNRS, Paris, France

**Keywords:** Diversity, Integration, Transnationalism, Solidarity, Belonging, Territory, Nationalism

## Abstract

Theoretical and normative approaches regarding the question of diversity and integration, such as multiucluturalims and interculturalims compete in an attempt to redefine citizenship and nationhood. Most analyses have been single-theory-oriented, leading to multiple, contested and controversial interpretations of integration and democratic public spaces.

Transnationalism raises the question of the limits of national public space and extends the concept of cultural integration beyond borders challenging the normative theories of multiculturalism and interculturalism bounded to national societies. Whatever the ideology and objective in the understanding of integration, states are confronted today with the transnational actions of activists who try to bypass states in order to reach a global perspective of their identification and action. Solidarity beyond borders involves a multilevel interaction between home and host countries and leads the states to develop strategies of integration – territorial and non-territorial – as a way of including identity issues developed in a minority situation into their political strategy to “re-territorialize” them. The objective then is to counter non-territorial solidarity expressed in global religious terms, mostly virtual, diffused by the Internet, which attracts the young generation, urging them to reject any or all national identification, to develop a new pride, a sense of community based on a global identification.

## Introduction

Theoretical and normative approaches regarding the question of diversity and integration compete in an attempt to redefine citizenship and nationhood. Multiculturalism, the most controversial, defined as the “politics of recognition” by Taylor (1994), has connotations of “tribalism” and “groupism” (Brubaker, 2002) and is perceived as a challenge to national unity. For Modood (2017), multiculturalism is the extension of the concept of national citizenship and of nationalism, and he suggests a “multicultural nationalism” as a way “to accommodate British Asian Muslims political assertiveness”. On the opposing side, the concept of interculturalism, according to Zapata-Barrero (2017), is “post multiculturalist”. It is “contact based” leading to a “mutual belonging” of nationals and non-nationals and to a “civic practice and citizenship”.

Both multiculturalism and interculturalism were developed in Canada as a result of the linguistic confrontation between French and English and the broader debates on a bicultural society. The official definition of multiculturalism by the Royal Commission on Multiculturalism gave political legitimacy to the concept through the constitutional definition of multiculturalism used in the Charter of Rights and Freedoms. This view was officially accepted as the fundamental characteristic of the Canadian State. In 2008, Taylor and Bouchard reported on the public perceptions regarding multiculturalism. They suggested interculturalism as a model of integration for the province of Québec and multiculturalism for Canada. Interculturalism, a “new formula of coexistence” (Bouchard, 2011) is designed to bring a dynamic perspective to the “defined national identity”. The argument is based on the importance of a dialogue among cultures as a basis for reciprocity, which leads to a cohesive society where solidarity includes the majority as well as the minority. In its definition, interculturalism is thus opposed to the recognition of separate groups’ assertiveness in the public sphere commonly attributed to multiculturalism.

## Concepts and contexts

Similar situations entail recourse to concepts that, used in different national contexts, require new definitions. In Europe, the Council of Ministers approved the White Paper on Intercultural Dialogue in 2008, which declares interculturalism as the basis for a European identity. This sentiment is reflected in the official motto “unity in diversity”. The idea is to combine the pluralistic and complex sense of belonging among individuals, groups, and peoples in order to construct a political identity that is purportedly European, or rather, to arouse identification with Europe as a new political space of action and demands. In different European countries multiculturalism corresponds to various situations according to the structure of the state and its recognition of regional and linguistic particularities. According to Kymlicka’s (1995) typology there are multinational states (states that are constituted of national entities that are defined in terms of language and territory, such as Spain, Belgium, Switzerland) and polyethnic states (with several ethnic communities born from immigration). In this latter category are France, Germany, the Netherlands where an important issue pertains to the settlement of post-colonial migrants – the majority are of Muslim origin – and their mode of integration, including the expression and organization of collective identities, their claims for representation and recognition, the role of religion, and the increasing influence of diasporic networks and the transnational politics of their home countries. In the public debate, multiculturalism marks the shift from temporary economic immigration to the permanent presence of immigrant populations. In Germany, it has become a way of making public opinion and politicians aware that “foreigners are here to stay”. In France, concerned with a democratic society governed by equal rights, discourses on a “multicultural society” elaborate on a democratic society governed by equal rights and ultimately attempt to augment public opinion for diversity as an inherent fact in any democratic society. In both countries Germany and France, multiculturalism acknowledges diversity and develops a narrative and a policy of integration that considers migrant as co-citizens.

Despite different narratives and normative perspectives, European countries have gradually converged on a sort of “applied multiculturalism”, regarding urban policy, education, and “politics of difference.” In France, the politics of difference involves the recognition of voluntary associations and their activities based on collective interest expressed in terms of identity. Empirical studies show that voluntary associations’ activities can be ranged from community building to individual social mobility (Kastoryano, 2002). On the other hand, a comparative study in Europe shows that integration policies promoting multiculturalism have not improved the economic situation for immigrant populations. In fact, they have contributed to their cultural and political marginalization (Koopmans, 2010). This conclusion is contradicted by a comparative study of nineteen European countries that demonstrates the positive effects of multiculturalism policies, especially on migrants’ political participation (Kesler & Bloemraad, 2010).

Great Britain is the European country where the normative value of the concept of multiculturalism is most elaborated. In 2000, Parekh published *The Future of Multi-ethnic Britain*, which expressed his hope of seeing a Britain that would simultaneously respect equality, difference, individual freedom and community cohesion: *“Britain should develop both as a community of citizens (the liberal view) and a community of communities (the pluralist view)*.” For Modood (2012), multiculturalism is based on the democratic values of liberty, equality, and fraternity and unity. Arguing against the assimilationist approach because of its defense of a historical national homogeneity, he believes that multiculturalism considers migrants as co-citizens within a pluralism in which all identities are respected. Moreover, political strategies that move in this direction in order to reshape the understanding of Britishness. Yet again in Britain, the 2001 Cantle Report denounced the “parallel lives” created through multiculturalism and pushed for a *“new framework for race and diversity based on interaction and positive values for diversity”* (Cantle, 2001). A “narrative” of “community cohesion” defended by interculturalism (Cantle, 2012) has been intertwined with the “normative” theory of multiculturalism and its political polices and outcomes.

Zapata Barrero (2017) maintains the normative strength of multiculturalism through his emphasis on equality. Interculturalism is also concerned with the inclusion of groups since they constitute a *“collective resource for the benefit of the collectivity”.* It suggests therefore a *“reformulation of the common public culture in terms of community cohesion”,* promotion of solidarity, and of a common public culture placing diversity in its center. He argues that such an approach fills what multiculturalism seems to have underestimated: *“contact and dialogue, and interpersonal relations between people from different backgrounds, including nationals and citizens.”* This claim implies that multiculturalism has led to the fragmentation of society into communities turned inward in their identity and distinct from the political community. Interculturalism, considered as a “post-multicultural period” by Zapata-Barrero, becomes an instrument to facilitate a sense of mutual belonging as defended by the Intercultural Cities Program of the Council of Europe (2008).

Modood (2017) regrets the lack of “academic engagement” and theoretical foundations of the concept and argues that interculturalism is essentially a variant of multiculturalism. He argues also that, contrary to what interculturalists pretend, dialogue among groups has always been at the heart of multiculturalism (Meer & Modood, 2012) and the most important aspect of the “politics of recognition” is “building a relationship of trust”; a value that interculturalists cannot neglect, since it is hard to conceive communication, dialogue and solidarity without trust. Is interculturalism misrepresenting multiculturalism as Modood claims?

It is difficult to draw normative boundaries between these two a priori opposing concepts. For Modood (2017), the central normative claim is that citizenship and national identity must be remade to include group identities that are important to minorities as well as majorities. He argues that *“this is double-aspected: the right to recognition of difference, to distinct cultural needs and provision but also the right to be included, to full membership, which includes the sharing of the national-public space or culture and in the sharing to remake it”.* He suggests a “multicultural nationalism” that is only civic in nature because it is based on the recognition of groups rejecting membership as ascriptive, as well as the concept of “Otherness that refers to Ethnicity”. French republican rhetoric couldn’t agree more. Zapata-Barrero (2016) sees interculturalism as fostering intercultural citizenship, and consequently it is seen as an important driver for a socialization process, of culture-making. Diversity is then an advantage and a resource to promote solidarity and reframe a common public culture.

What is the difference between a multicultural nationalism and intercultural citizenship - both aim for a civic public space leading to a new dynamic of national identity - other than a narrative difference? The problem that emerges in the elaboration of both concepts is the use of categories. Modood acknowledges the normative significance of the majority, emphasized by interculturalists, but also stresses a “new majority” that would not be the predominant group. Would that majority be “recognized” on an equal basis with other groups that claim state recognition and representation in order to achieve equality? How do we imagine a majority without the historical process of a nation building and the pressure for defining a national identity? Despite the idea of a common past, national identity, similar to all identities, is redefined according to the expectations of the social groups constituting it as its relation to other nations. Would the influence of internal and external dynamics bring the majority – an important part of the historical formation of the state as well – to the same level as minority groups claiming state recognition? Does equal citizenship, on the basis of rights and duties, erase the historical trademark of the majority?

Multiculturalism as history in the making requires new compromises and negotiations among states and groups (Kastoryano, 2002). The terms of the compromise take into consideration the dynamics in the definition of national identity, but it is difficult to imagine that the majority will be a group as any group looking for equal recognition. As for the elaboration of interculturalism, what does Zapata-Barrero (2017) mean when he defines the concept as *“contact base communication and relationship among people from different backgrounds, including national citizens”*? A distinction between “national citizens” and “other citizens” contradicts the very understanding of civic citizenship and the principle of equality. Multiculturalism is not only about “the inclusion of immigrants into the mainstream by respecting their differences” and “the protection of their rights”, but it also relates to all discriminated groups because of their difference (Phillips, 2007). The confusion comes from legal, categorization such as citizen and immigrant, national and non-national, citizen and non-citizen, and representations of “Otherness” in which immigrants are placed. Such categories are likely to maintain “identity boundaries” even though “the added value” of interculturalism, according to Zapata-Barrero, is communication and the importance of a civic national narrative.

Such a categorization refers to the multiculturalism debate developed after 9/11, as asserted by Zapata-Barrero (2017), the Muslim population as an explicit target. The use of the concept has shifted then from the political, cultural and social sphere to the sphere of values, with regard to liberalism, and questions the “democratic defense of cultural diversity within a universalist perspective”. He also stresses that the successive terrorist attacks of the early 2000’s have associated the term with security issues and placing Islam at the center of concerns. According to Kymlicka (2012), the regression, of multiculturalism is due to perceived threats hanging over border security, human rights and economic prosperity. To ensure its success, ethnic relations must be “desecuritized.” That should also be the success of interculturalism.

## Interculturalism: an idea, a policy or a narrative?

In Europe, the interculturalist narrative is, *“a policy narrative that existed in earlier practice.”* Zapata-Barrero (2017) gives the example of Barcelona that, in 1997, opted to call its policy *“intercultural as a result of dissatisfaction with the existing multicultural/assimilationists models in Europe”*, a policy that focused from the beginning *“on promoting contact rather than separation”.* In France, the “idea” was expressed as “intercultural policy” in the 1970s and was a part of education policy focused on the “value of reciprocity and exchanges.” This policy has led to a “political measure to install positive interaction and comprehension among students from different cultures” (Kerzil, 2002), along with what was called then “classes of integration” for the children of immigrants. In 1973, the introduction of a social policy called “intercultural education” was described as a way for *“French children to learn about other cultures in order to live better together”.* The intercultural educational policy appears as a sort of pragmatic policy of *“mutual acceptance at school and in the neighborhood”*. However, research based on the experience show that the policy was limited in practice to “everyday diversity” and faced problems of accomplishment.

The school as a site of intercultural policy was obviously not a coincidence. It was also not a coincidence when the school was involved in the 1989 the site of the headscarf affair that shook French society. Political and academic debates focused precisely on the function of the school as the institution that most clearly embodies the “national ideology”. Was the affair a sign of the failure of “intercultural education” policy – a policy that referred to food, language, history of the country of origin, and “everyday diversity” – but did not take into consideration religion as a part of “cultural difference”? The headscarf affair not only showed the limits of intercultural education policy, but also situated secularism – *laïcité* – at the core of the French Republic as its fundamental value alongside a discourse on individual liberty, religious freedom, human rights, and the emancipation of women. Nevertheless, the never ended defining “cultural difference” in terms of religion, Islam, perceived as a permanent difference, contradicts integrative value of laïcité. *Laïcité*, as a constitutional value and as a principle representing the neutrality of the state regarding religion, could have been the basis for equal representation and recognition of all faiths (Art. 2 of the Constitution) instead of emerging against Islam as an “official religion of France.” It seems that *laïcité* has missed its chance to truly be a universal principle through the incorporation of Islam into the public space, a religion that has been outside the history of the relationship between church and state that shaped French national character.

*Laïcité* is an element of national history related to the institutional setting of religion and its contextual accommodations. The separation of church and state confers institutional legal status on the Catholic clergy, the Protestants of the National Federation of Protestant Churches of France, and to the Jews governed by the Consistory created under Napoleon. With Islam as an emerging religion, the extension of institutional recognition for equal representation has been defined as a pluralist promotion of diversity. Furthering multiculturalism for Modood (2017) is to extend the privileges of the Church of England, an “institutional figure of England’s and British national identity”, to other faiths in order to achieve a multicultural nationalism. Non-denominational state schools should also include compulsory religious education of all faiths as a part of a national curriculum. It seems that two different national histories, different relationships between Church and State, lead to different perspectives of equal representation of religion within the institutional settings of each nation, and the understanding of the public sphere (see also Wimmer & Schiller, 2003).

While multiculturalism focuses on a national level questioning the national identity, interculturalism emphases the local level because of the geographical and physical proximity among groups that facilitate dialogue and exchanges. Such an assumption suggests a social link between separate communities settled in one locality. But the social reality of concentrated and/or segregated neighborhoods, ghettos, or *banlieues* in France, or Londonistan in London, or Kreutzberg in Berlin, show that these urban settings have become spaces of tensions among communities. Ethnic grouping in these neighborhoods reflects the failure of integration policies and urban policies, an evolution that nourishes the discourses on the failure of multiculturalism but not guarantee the success of interculturalism.

## Challenge to multiculturalism and to interculturalism: transnational nationalism

The greatest challenge to the current debate on multiculturalism and interculturalism as a set of policies is the power of transnationalism. On a macro and/or micro level, both, multiculturalism and interculturalism are developed as a theory, a narrative and political paradigm in response to the management of cultural diversity within the nation-state in order to redefine citizenship, nationhood, as well as local and national solidarity. Transnationalism, however, has been developed as an experience of migrants and minorities, settled in different national societies interacting with each other beyond borders. Increasing mobility and the development of telecommunication technologies have intensified such trans-border relations and mobilizations, and have participated in the elaboration of a transnational solidarity and identification. Such an evolution is the result of intense and complex ongoing ties that migrants uphold with their country of origin and the cultural, social, economic, political and ideological transfers that occur between the departure and the receiving country and beyond. These multiple levels of participation are perceived as a challenge to the founding principles of nation-states with regard to territoriality, citizenship, and membership to a single political community.

Transnationalism brings to light multiple membership and multiple loyalties – crystallized around dual citizenship – which becomes for immigrants a way to maintain an identity rooted in the home country. Citizenship becomes then an entitlement within the country of residence. This development is used to form “diaspora politics” as a means to maintain the loyalty of the citizens “abroad” as well as to extend its power beyond territories. Turkey and Morocco, where national and religious identities are combined are the most active in such transnational politics. The main objective is to oppose the strategy of international organizations that promote global Islam by re-territorializing and re-nationalizing their belonging expressed in terms of religion and to control the citizenry and loyalty abroad as a resource for the transnationalization of the state (Kastoryano, 2016).

Home states attempt to influence integration of the state (both states) and transnational communities into a global space as a way to compete with transnational communities in their engagement of the process of globalization. States also attempt to control transnational actions, which by definition intend to bypass the state. Transnational politics reflect therefore the changes in the “paradigms of integration” as formulated by Zapata-Barrero (2017), and the perception of migration, linking increasingly the question of identity and participation to the question of security. Receiving countries are driven to collaborate with home countries to insure migrant integration.

In this configuration, politics of integration are not a single state policy. States (home and host) cooperate for integration to insure re-territorialization of globalized identities with transnational action. In the case of transational Islam, these actions are promoted by international organizations through images, symbols and speeches, founded on a religious and/or ideological identification. As for Muslims in Europe, they express their attachment to the country of settlement in terms of citizenship and rights. They express also their loyalty to the country of origin in terms of emotions and identity. Being a “Muslim minority in Europe” has created a new “imagined global diaspora” and that brings a third dimension based upon a religious identification that is transnational both in essence and definition, and which goes beyond Europe. Werbner (2002) shows how “*imagining their different diasporas, local Pakistani tended to position themselves imaginatively as the heroes of global battles”, and argues that “diasporas are transnational communities of co-responsibility”.* In an “imagined global diaspora” where individuals and groups and transnational communities are connected in global networks, the traditional diaspora loses its territorial bases, in which home is an imagined place to express precisely “co-responsibility” without a territorial reference as “home”, more over so for the younger generation who has an abstract image of the home country of their parents.

Thus, transnational politics of both communities and states create new configurations of nation and nationalism, of territory and power, in globalization. Communities, based on cultural, ethnic, and religious identifications and recognized by states that increasingly rely on transnational solidarities have sparked new upsurges of nationalism. States on the other hand expand their nationalism to maintain the “power” of incorporation and citizenship, in order to re-territorialize identities here and/or there.

The multiplicity of identities – national, religious, ethnic or linguistic – that are fragmented yet represented in such a structure, is “re-centralized” in a non-territorial way around a rhetoric of mobilization. The internal diversity of the transnational community is “re-centered” also around norms and values diffused by European supranational institutions and through the process by which these same institutions give the diversity a legitimacy on the international stage. Such legitimacy is driven via an inclusive discourse developed by transnational activists founded on human rights and the fight against racism or any other form of social, political and cultural exclusion. The same diversity is “re-centered” also around being a Muslim minority to provide a basis for a narrative of belonging to a global Muslim community, which is then re-interpreted in such a way as to reframe all the internal diversity into an “imagined transnational community”, or an imagined global diaspora, or even an imagined global nation that defines itself as a cultural nation. The unity of the transnational community is sustained by the desire to belong to a “people” through a process of nominal appropriation of its actions and discourses, a sense of participation in its “destiny”.

Although transnational communities have emerged as a logical next step to cultural pluralism and to identity politics, the recognition and representation of groups has led them at the same time, to redefine solidarities on a transnational level with new subjectivities, accompanied by the imagined geography of the “transnational nation” (Kastoryano, 2007). Its territorial frontiers are not disputed. On the contrary, its non-territorial borders follow the web of formal and/or informal networks transcending the boundaries of national territories and engendering a new means of invisible and unenclosed territorialization. This development also produces a political community in which the individual’s actions inside the network become axioms of a transnational non-territorial nationalism that seeks to strengthen itself by employing discourses, symbols, images, and objects.

The development is inscribed in a global space that *does not translate* but rather *produces* an identity and generates a mode of participation across borders, as shown by the engagement of actors in the consolidation of transnational solidarities through action, and mobilization. By reflecting on the state “deficiencies” regarding human rights or citizenship, the actors seek to channel the loyalty of individuals from a territorialized political community towards a non-territorialized political community to redefine the terms of belonging and allegiance to a “global nation”. This global nation finds comfort in the rhetoric of diffused unity thanks to modern technology, producing a single *language* – images – or a single *langue* such as English as a medium of participation in internet sites and email exchange. Regarding Islam on the Internet, see the work of (Roy, 2006). It is expressed both on an everyday basis and in long-term political goals; it is developed in different domains and territories - real or symbolic - trying to re-establish social relations and a common identification. This identification is seen in the violence perpetrated in the name of a cause that directly or indirectly affects an Islam which is perceived as a “global victim”, an image reinforced by the rhetoric of Western humiliation and domination and propounded by what Leiken (2012) calls “Angry Muslims”.

Such an evolution challenges the multicultural nationalism “imagined” by Modood (2017). Transnational nationalism focuses on an “invented”, abstract identification with an “imagined global community” fuelled by external events such as wars, conflicts in other countries, and colonial relations yielding to an expression of local and transnational autonomy. Diaspora politics of home states, in their objective to re-territoiralize globalized identities, come to “re- ethnicize” them, which in return affects their attitudes in the country of settlement. It would be interesting to empirically determine how transnational actors perceive multiculturalism and how diaspora politics affects their involvement in multiculturalism as policy and as a discourse. In any case, opposition to normative multicultural nationalism and the emergence of an “imagined” transnational nationalism, old fashioned, ethno-cultural nationalism, renamed populism, started to proliferate all over Europe. Their rhetoric, political program, and capacity to mobilize public opinion nourish exiting discourses on the failure of multiculturalism and revitalize state nationalism based on the protection of territorial boundaries and national identity. The “return” of nationalism in certain European countries has used the migrants’ crises attributing migrants and/or asylum seekers a transnational solidarity perceived as a threat to national sovereignty and even though the phenomenon has nothing to do with transnational networks and group membership.

How do transnational practices of individual and groups affect interculturalism and its focus on dialogue and the mutual belonging on local level? Zapata-Barrero (in press) sees in these two phenomena an overlapping situation. A group involved in transnational action and mobilization, that sees in transnationalism an “identity refuge,” may or may not communicate with other groups. This perspective involves two levels of analysis which are in fact interconnected, and two interdependent modes of identification: local (territorial) and global or transnational (non-territorial). The juxtaposition of communities as a source of tension in some localities might not create an identification with “the place” because of the strength of transnational networks.

These are empirical questions. They require a field work to see how the spiraling of violence in the Middle East, the 9/11 (11 September 2001) attacks, the wars in Iraq and Syria, and many other international events that have contributed to producing both heroes and victims among the young, influence their speech and their action as a sort of de-territorialized revenge that is localized in urban areas. Violence also allows a form of territorialized and ethicized collective expression to develop, re-centering the diversity of the de-localized population around new subjectivities nourished by unifying discourses that seek to re-define solidarity and build a coherent whole. These references produce an identity that is not linked to the immediate space but to a non-territorial community, which becomes a refuge for a young generation that is looking for a cause and identification in action. The process gives rise to the formation of a transnational identity as inspiration for political action and as an instrument for cultural and religious purposes beyond national borders. Only empirical research will show individual and group dynamics, as well as international influences that will affect both multiculturalism and interculturalism. The question is how “diaspora politics” of home countries and/or international organizations and their local level networks confront, cooperate, or compete with urban politics and the management of diversity? An empirical approach can help to clarify the nature of relations among multiple, overlapping and conflicting conceptions of cultural integration and then develop a normative approach.

## Conclusion

The management of diversity has led to the normative theories of multiculturalism, interculturalism, inspired by Canadian political theory and by Canadian reality. Transposed into European context, each approach has its own conception of national unity, equality and solidarity. Most analyses have been single-theory-oriented, leading to multiple, contested and controversial interpretations of integration and democratic public spaces.

Transnationalism raises the question of the limits of national public space and extends the concept of cultural integration beyond borders challenging the normative theories of multiculturalism and interculturalism bounded to national societies. Whatever the ideology and objective in the understanding of integration, states are confronted today with the transnational actions of activists who try to bypass states in order to reach a global perspective of their identification and action. Solidarity beyond borders involves a multilevel interaction between home and host countries and leads the states to develop strategies of integration – territorial and non-territorial – as a way of including identity issues developed in a minority situation into their political strategy to “re-territorialize” them. The objective then is to counter non-territorial solidarity expressed in global religious terms, mostly virtual, diffused by the Internet, which attracts the young generation, urging them to reject any or all national identification, to develop a new pride, a sense of community based on a global identification.

Transnationalism engenders a distinct sense of non-territorial nationhood and generates confrontations among multiculturalist and interculturalist perspectives. They all give rise to arguments and policies that reinforce the new classical version of state nationalism. One of the challenges of globalization is to find a new source for new normative theories with regard to diversity.
